# Prevalence of and risk factors for long COVID following infection with the COVID‑19 omicron variant 

**DOI:** 10.3892/mi.2025.216

**Published:** 2025-01-24

**Authors:** Isao Moritani, Kenji Yamanaka, Tai Nakamura, Junichiro Tanaka, Keigo Kainuma, Masakazu Okamoto, Tomoko Ieki, Hideo Wada, Katsuya Shiraki

**Affiliations:** 1 Department of General Internal Medicine, Mie General Medical Center, Yokkaichi, Mie 514-8561, Japan; 2 Sasagawa Clinic of Gastroenterology, Yokkaichi, Mie 510-0961, Japan; 3 Nakamura Heart Clinic, Yokkaichi, Mie 510-0892, Japan; 4 Tanaka Internal Medicine Clinic, Yokkaichi, Mie 510-0942, Japan; 5 Kainuma Clinic, Yokkaichi, Mie 510-0892, Japan; 6 Department of Infection Control, Mie General Medical Center, Yokkaichi, Mie 514-8561, Japan; 7 Research Center, Mie General Medical Center, Yokkaichi, Mie 514-8561, Japan

**Keywords:** COVID-19, long COVID, omicron variant

## Abstract

The aim of the present study was to clarify the current status of persistent symptoms following omicron variants of COVID-19 [long COVID (LC)] and the risk factors associated with the development of LC. For this purpose, a cross-sectional survey of patients with COVID-19 treated at the authors' hospital and at four associated outpatient clinics was conducted. Questionnaires about a post-infection condition were sent by post to 3,399 patients. A comparison was made of patients infected when the omicron variant was prevalent (omicron group) and patients infected prior to that (pre-omicron group). Valid responses were received from 1,113 (32.7%) patients. The percentages of patients in whom some type of symptom or sequelae continued after 1, 3 and 6 months were 44, 37 and 28%, respectively, in the pre-omicron group, and 20, 12 and 9%, respectively, in the omicron group. The percentages were significantly lower in the omicron group. In the multivariate analysis of risk factors for LC at 3 months, significant risk factors were hospitalization [odds ratio (OR), 1.910; P<0.01] and old age (OR, 1.120; P<0.05). Conversely, the incidence of LC was lower with vaccination (OR, 0.830; P<0.01) and in the omicron group (OR, 0.490; P<0.01). In the omicron group, underlying disease (particularly emphysema, bronchial asthma, rheumatoid or collagen disease and hypertension) and hospitalization were significantly associated with LC (P<0.01). On the whole, the present study demonstrates that the incidence of LC was >10% even in the omicron group. Patients who required hospitalization and patients with underlying conditions require careful attention for the development of LC.

## Introduction

COVID-19 is a multisystem disease with severe symptoms following infection with the severe acute respiratory syndrome coronavirus 2 (SARS-CoV-2). With the spread of COVID-19, some symptoms persist beyond recovery from the acute stage and become chronic (1,2). Symptoms following illness due to COVID-19 (sequelae) include both those that persist from the period soon after COVID-19 was contracted and new symptoms that appear after recovery. These symptoms continue despite the disappearance of infectiousness following COVID-19. A Delphi consensus has been used as a definition of long COVID (LC) as a post-COVID-19 condition. According to this consensus definition, LC is a post-COVID-19 condition that occurs in individuals with SARS-CoV-2 infection, usually 3 months from onset, with symptoms that last for at least 2 months with no other explanation (1). These symptoms include, but are not limited to, disorders of the respiratory, cardiovascular, musculoskeletal, skin and nervous systems, and LC is considered a multisystem disease with a wide range of symptoms. In reports to date, the percentage of patients with post-COVID symptoms has been considered to range from 10% to ~60% (3-6).

Typical LC presents with fatigue/malaise, joint pain, myalgia, cough, sputum, dyspnea, chest pain, alopecia, memory disorders, loss of ability to concentrate, headaches, depressed mood, olfactory disorder, dysgeusia, palpitations, diarrhea, abdominal pain, sleeplessness, muscle weakness and skin rash. Previous studies have noted that LC may severely affect the daily lives of affected individuals (2-5,7-9). The precise pathogenic mechanisms of the condition are not yet fully understood; however, multiple factors, such as direct organ damage, autoimmune reactions, inflammatory reactions and mental disorders from SARS-CoV-2 infection, appear to be involved (10).

Reports of post-COVID-19 symptoms are increasing globally; however, survey results differ greatly depending on differences in settings for the patient population, including patient background, particularly the presence or absence of a definitive diagnosis, the age of the infected person, the severity of the disease, and whether they are inpatients or outpatients. Bias may also occur, for example, in data collection methods. Differences may also arise from the COVID-19 infection strain. The omicron strain is generally considered to have fewer sequelae than earlier strains (11-14).

Moreover, the weakness that is sometimes observed following the treatment of severe acute diseases not limited to COVID-19, underlying conditions from before a person became ill with COVID-19, and the mental and physical effects from changes in lifestyle due to the pandemic may render the clinical picture of post-COVID-19 symptoms more complex and difficult to analyze (15).

In general, a number of these post-COVID-19 symptoms tend to improve with time (16); however, the transition of these symptoms over a long-term period, the extent to which there are differences from the different infecting strains, and the exact risk factors involved in the development of post-COVID-19 symptoms are not yet fully understood.

In the present study, to clarify the actual incidence of and risk factors for LC in patients with COVID-19 with both pre-omicron variant and omicron variant in the community, and to establish strategies for the prevention of LC, a questionnaire survey of patients with COVID-19 in multiple facilities, including a regional hospital and associated clinics, was conducted.

## Patients and methods

### Patients

The present study was a multicenter, cross-sectional study conducted at one inpatient hospital (Mie General Medical Center, Yokkaichi, Japan) and four affiliated community clinics (Sasagawa Clinic, Nakamura Heart Clinic, Tanaka Internal Medicine Clinic and Kainuma Clinic, Yokkaichi, Japan). The hospital is a regional hospital that accepts a wide range of patients from mildly to critically ill. All participants were from the almost same region, and it was assumed that there were not marked differences in social backgrounds in this study period. The participants were individuals diagnosed with COVID-19 up to January, 2023 at the participating institutions. Their ages ranged from 15 to 89 years, and they were patients who had suffered from COVID-19 at least 6 months prior to the time they responded to the questionnaire. No other special exclusion criteria were set. Questionnaires were sent to 3,399 patients. They included not only Japanese individuals, but also foreign nationals residing in Japan.

### Analysis methods

Questionnaire requests were sent to participants by post. Responses were received either on mail paper or over the internet. Subjects were registered by responding to the questionnaire. The present study was reviewed and approved by the Ethics Committee of the Mie General Medical Center (O-0142). The survey included detailed explanations of the purpose of the study's, and responses implied informed consent. Minors provided consent to complete the survey with the approval of their parents or guardians.

According to survey results in Japan, following the outbreak of COVID-19, the variants of the COVID-19 virus were the alpha and delta variants for the period to December, 2021; these variants were then rapidly replaced in nearly all cases by the omicron variant (17,18). Therefore, in the present study, patients who developed COVID-19 after that time constituted the omicron group, whereas those infected prior to that time constituted the pre-omicron group.

### Questionnaire

The questions on the questionnaire could be answered fairly easily. They included questions on sex, age, smoking and drinking status, pregnancy, underlying conditions, period of infection, number of times they had received the novel coronavirus vaccine prior to infection, symptoms during the time of infection, treatment during the time of infection, whether there were sequelae after infection, treatment for the sequelae, symptoms and duration of the sequelae, and others. Choices for symptoms experienced as COVID-19 sequelae were made from a list of generally reported sequelae, but they could also be described freely.

### Statistical analysis

Individual questions on whether a sequela had developed after >1 month were evaluated. For binary questions, Fisher's exact tests were performed. The Cochran-Armitage test was used for age, number of infections, number of vaccine doses, and infection period. Factors with significant associations in the univariate analysis were included in the multivariable analysis. EZR software was used for the analysis (19). The level of significance was 5% and a P-value of P<0.05 was considered to indicate a statistically significant difference.

## Results

### Patient characteristics

**Figure 1:**
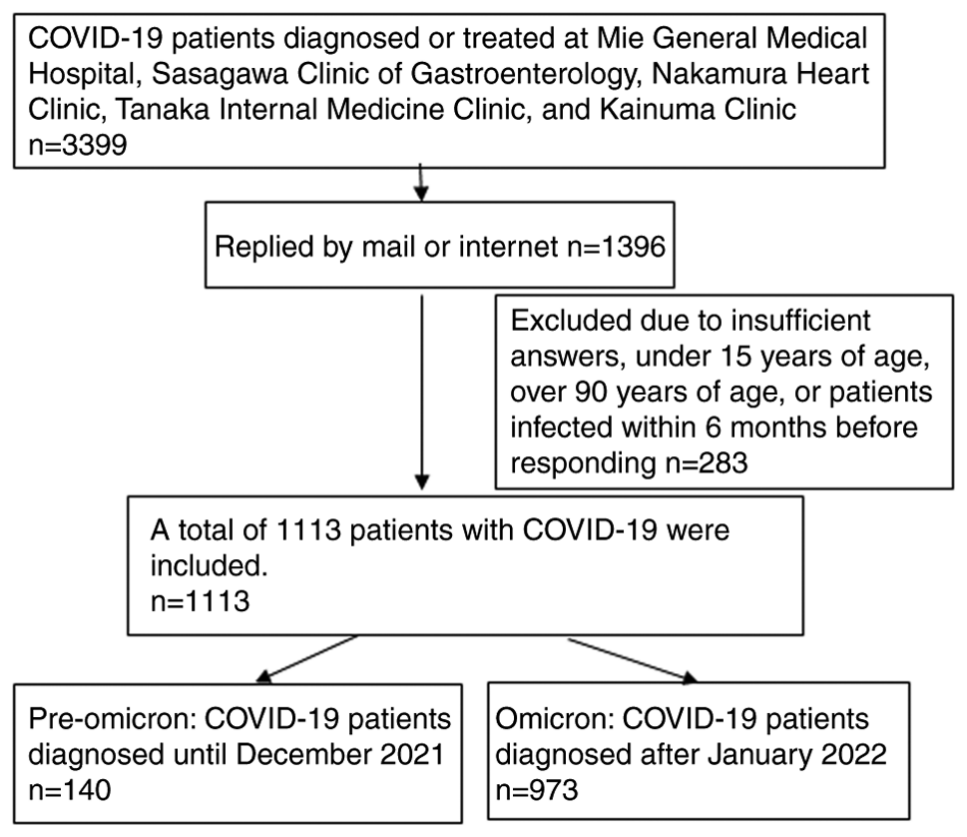
Flow diagram of the screening process used for the patients with COVID-19 in the present study.

Responses were received from 1,396 individuals, with valid responses from 1,113 individuals (32.7%) (Fig. 1). Age was distributed over a wide range in each age group, with 104 individuals in their teens, 137 in their twenties, 138 in their thirties, 175 in their forties, 168 in their fifties, 176 in their sixties, 145 in their seventies, and 70 in their eighties. There were 483 males and 630 females. Of the female respondents, 36 (5.7%) were pregnant at the time of infection (Table I).

There were 700 participants (62.9%) in the never smoker group, 325 (29.2%) in the former smoker group and 88 (7.9%) in the current smoker group. As regards alcohol consumption, there were 563 participants (50.6%) in the never drinker group, 333 (29.9%) in the social drinker group, and 217 (19.5%) in the group who consumed alcohol two or more times per week. For underlying diseases, a list of options was shown, in addition to which they could be described freely. An underlying disease of some kind was reported by 509 participants (45.7%).

The omicron group included 973 (87.4%) patients, and the pre-omicron group included 140 (12.6%) patients. The number of vaccine doses was 0 for 214 participants (19.2%), 1 for 18 participants (1.6%), 2 for 235 participants (21.1%), 3 for 378 participants (34.0%), 4 for 203 participants (18.2%) and 5 for 65 participants (5.8%). The number of participants who were hospitalized was 204 (18.3%). Of these, 50 (4.5%) participants were administered oxygen. The number of smokers was significantly higher in the omicron group than in the pre-omicron group. By contrast, the numbers of participants with diabetes mellitus, a history of hospitalization while ill with COVID-19, and those who received oxygen were significantly lower in the omicron group (Table I).

### Symptoms during initial infection

Symptoms reported by at least 20% of cases during infection were fever (78.4%), a sore throat (54.4%), cough (50.8%), fatigue (45.6%), headache (27.8%), phlegm (23.2%) and nasal discharge (22.1%). Sore throat was significantly more common in the omicron group, whereas dysgeusia, olfactory disorder, dyspnea and sleeplessness were more common in the pre-omicron group (Table II).

### Symptoms of LC

**Figure 2:**
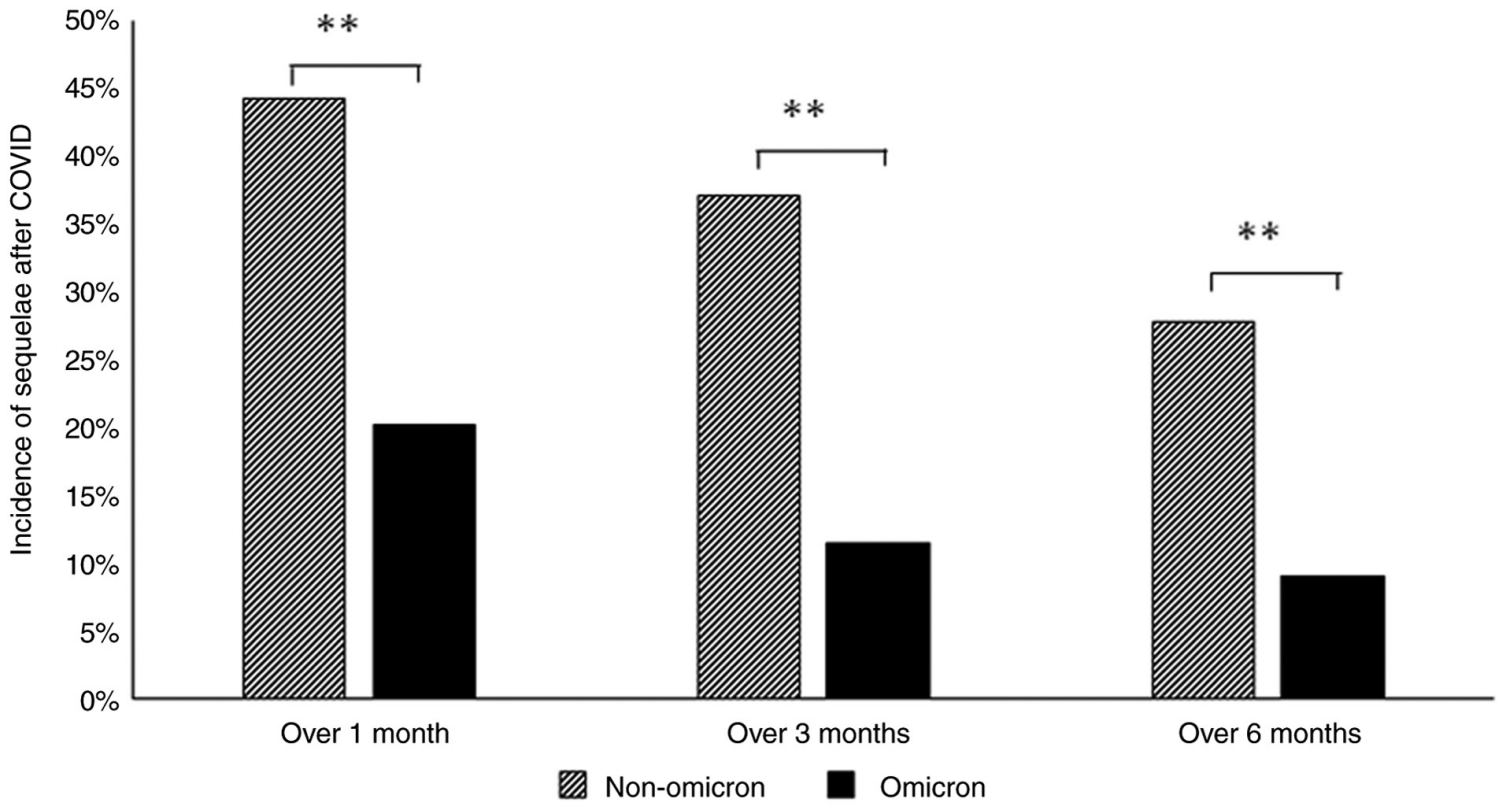
Incidence of sequelae following COVID-19 infection in the pre-omicron and omicron groups. ^**^P<0.01.

The percentages of patients in whom some type of sequelae persisted at 1, 3 and 6 months after the infection were 44, 37 and 28%, respectively, in the pre-omicron group, and 20, 12 and 9%, respectively, in the omicron group. The percentages were significantly lower in the omicron group (Fig. 2). Even at 3 months, which is defined as LC, >10% of the patients in the omicron group were symptomatic. Symptoms as sequelae after 3 months in the pre-omicron group were, in order of frequency, fatigue, alopecia, loss of concentration, dyspnea, olfactory disorder, dysgeusia, muscle weakness, memory disorder, headache, cough and others. In the omicron group, the frequency of all LC symptoms was lower than that in the pre-omicron group; in particular, the frequencies of alopecia and olfactory disorder were low (Table III).

### Risk factors for LC

The factors related to LC symptoms at 3 months after infection were analyzed. In all subjects, significant risk factors in the univariate analysis were underlying disease (P<0.001), two or more vaccine doses (P<0.001), oxygen inhalation (P<0.001), hospitalization (P<0.001) and the pre-omicron variant (P<0.001). Significant differences were also observed in age, with the highest number of LC cases (23.81%) observed in patients in their fifties, with a tendency to be lower in younger groups. No significant differences were observed in sex, smoking status, drinking habits, antiviral drug history and pregnancy (Table IV). In the multivariate logistic regression analysis, each factor which exhibited a significant difference in the univariate analysis, hospital admission [odds ratio (OR), 1.950; P<0.01] and age (OR, 1.160; P<0.01) were found to be significant risk factors. Conversely, the incidence of LC was significantly lower with vaccination (OR, 0.831; P<0.01) and in the omicron group (OR, 0.506; P<0.01). Diabetes and oxygen inhalation did not exhibit any significant difference in this multivariate analysis (Table V).

Subsequently, further investigations of LC in patients in the omicron group were conducted. The incidence of LC was significantly higher in patients with an underlying disease (P<0.001) and those who were hospitalized (P<0.01) (Table VI). In the multivariate logistic regression analysis for the type of underlying disease, emphysema (OR, 6.66; P<0.001), bronchial asthma (OR, 5.13; P<0.001), rheumatoid/collagen disease (OR, 3.31; P<0.05) and hypertension (OR, 1.77l P<0.05) were significant risk factors for LC (Table VII).

## Discussion

In the present study, a questionnaire survey of community-dwelling patients with COVID-19, including patients admitted to the hospital and those observed as outpatients in multiple institutions, was conducted. The prevalences of symptoms and sequelae were investigated, comparing cases that occurred during the omicron epidemic period (omicron group) and those that occurred earlier (pre-omicron group).

Of the symptoms at onset, there were significantly more cases of sore throat in the omicron group and significantly more cases of dysgeusia and olfactory disorder in the pre-omicron group. In a previous study, loss of smell and infiltration of the lower respiratory tract were less common in participants infected during the omicron pandemic than in those infected during the delta pandemic (20). In addition, sore throat was known to have been more common in patients during the omicron pandemic than during the delta pandemic. Furthermore, the hospitalization rate was reported to have been lower during the omicron period than during the delta period (20). The omicron variant is also markedly more transmissible than previous variants; however, in groups that have been vaccinated, the severity has been shown to be lower (21). A similar finding was observed in the present study. These findings suggest that different SARS-CoV-2 subtypes have different associations with symptoms and severity, and they also affect the frequency of LC (15).

In line with the definition of LC, in the present study, sequelae of some kind were confirmed in 37% of the pre-omicron group and 12% of the omicron group at 3 months. As for the prevalence of LC, there is a possibility of a lower risk of developing LC symptoms when infected with the omicron strain than when infected with the delta variant or other earlier variants (7,11,15). However, it should be noted that these studies were historical case-control studies.

The results of the present study are in agreement with these reports. That is, in the area in which the study was conducted, initial symptoms and the frequency of LC were low during the omicron pandemic. Of the LC symptoms, numerous patients experienced fatigue in particular; this was observed in >10% of patients in the pre-omicron group, and in only ~3% of the omicron group. A number of other symptoms, for example, loss of concentration, dyspnea, muscle weakness and memory loss, had lower frequencies in the omicron group; however, the frequencies of alopecia and olfactory disorders in particular were markedly lower in the omicron group. In previous studies as well, tiredness and fatigue were the LC symptoms with the highest frequencies (15,22-25). Fatigue is observed at even 100 days following the initial symptoms of acute COVID-19. Moreover, in syndromes, such as acute respiratory distress syndrome, it has been observed that more than two-thirds of patients report clinically significant fatigue symptoms after 1 year (3,26). The symptoms observed in patients who have suffered from COVID-19 are considered to resemble those of chronic fatigue syndrome, and exacerbations of severe incapacitating fatigue, pain, neurocognitive disability, compromised sleep and symptoms suggestive of autonomic dysfunction are also observed (3,27). The etiology of neuropsychiatric symptoms in patients with COVID-19 is complex and multifactorial. These symptoms may be related to the direct effect of the infection, as well as to cerebrovascular disease (including hypercoagulation) (3). In fact, the loss of concentration, memory disorder, sleeplessness, depressed mood and various neuropsychiatric symptoms were also reported in the present study, and measures for their diagnosis and treatment are desired.

The question remains regarding which types of patients are at a high risk of developing LC. In the present study, underlying disease, the number of vaccine doses (≤1), oxygen administration and hospitalization were related to the risk of developing LC. By age, the highest numbers of patients were in their fifties. In previous studies, an older age, the female sex, a high body mass index, complications, hospital admission or a large number of symptoms in the acute stage and disease severity were identified (4,9,10,24,28-29). In a previous study on adults in the UK, the female sex, an older age, obesity, smoking, vaping, hospitalization with COVID-19, poverty and being a healthcare worker were associated with a higher probability of persistent symptoms following COVID-19 infection (30). An Asian ethnicity was associated with a lower probability (30).

In the present study as well, which is considered to reflect the infection status in a region in Japan, the risk of LC was similarly increased with more severe symptoms in the early stage. This suggests that attention should be paid to LC in individuals with factors for more severe disease and patients with severe symptoms in the early stage. The risk of developing LC was also higher in patients with the underlying diseases, such as hypertension, diabetes mellitus, hyperlipidemia, bronchial asthma, emphysema, chronic kidney disease, hypertension and rheumatoid or collagen disease. These are also factors related to a greater severity of COVID-19(31), and they are considered to be related to LC.

Suggested mechanisms for LC include direct tissue damage from SARS-CoV-2 infection, constitutive inflammatory responses including autoimmunity and microvascular coagulation disorders with endothelial dysfunction (2,32). In addition, SARS-CoV-2 is neurotropic, and viral RNA and protein can be detected persistently in multiple organs including the brain, suggesting deep involvement in the pathology (33,34). The consideration of LC as a systemic disease, in addition to viral eradication in the early stage, and the handling of immune and coagulation disorders may also be required.

Individuals with a history of ≥2 vaccine doses in the present study had a lower risk of developing LC. In fact, this is supported by evidence which indicates that vaccination prior to infection lowers the risk of developing LC (35). Individuals who have had two vaccine doses have also been reported to have a significantly lower likelihood of developing long-duration symptoms (≥28 days) than unvaccinated individuals (10). In individuals who are vaccinated prior to acute infection or after infection, the risk of LC symptoms, such as sleeping disturbances, myalgia, renal injury and cognitive deficits has been reported to be lower with vaccination, and there is increasing evidence to suggest that lasting improvements are observed following a second vaccine dose (10,36). These findings suggest that vaccination contributes to mitigating the development of LC and symptoms; however, further clinical research on the effects of vaccination is required.

In addition to vaccines, the control of underlying disease symptoms is also considered to be critical in preventing LC. Early antiviral therapy has been reported to be associated with a decreased risk of developing LC, associated hospitalization and mortality. Therefore, early antiviral therapy is recommended for individuals who are at risk (37,38).

The present study has several limitations and biases, which should be mentioned. These include the fact that there are two methods of data collection, the fact that the infecting strain was determined according to the time of infection, the fact that the effect of treatment was not taken into account, the relatively low response rate, the non-response bias, and the fact that the accuracy of the answers was not guaranteed. Furthermore, the sample size may not have been sufficient to reach conclusions regarding the risk of developing LC. It was also not possible to objectively evaluate each symptom.

In conclusion, in the present study, a questionnaire survey of Japanese community-dwelling individuals who had been ill with COVID-19 was conducted. LC was found in >10% of the omicron group, although this was lower in patients in the pre-omicron group. These results demonstrate that this could have a notable impact on healthcare in the community. Risk factors for LC were found to include disease severity in the acute stage and underlying diseases, especially pulmonary diseases. Research on this topic is associated various issues in terms of methodology, and there is marked variability between studies in risk factors, definitions, follow-up duration and other matters. However, a notable number of patients require long-term care following infection even with the omicron variant. Clinically, it will be crucial to establish a medical strategy by predicting the progression to LC based on the background characteristics of patients with COVID-19.

## Figures and Tables

**Table I:** Background characteristics of the patients with COVID-19 in the present study.

Characteristic	All (n=1,113) (%)	Pre-omicron (n=140) (%)	Omicron (n=973) (%)	P-value
Age group				0.256
Teens	104 (9.3)	4 (2.9)	100 (10.3)	
Twenties	137 (12.3)	10 (7.1)	127 (13.1)	
Thirties	138 (12.4)	13 (9.3)	125 (12.8)	
Forties	175 (15.7)	25 (17.9)	150 (15.4)	
Fifties	168 (15.1)	28 (20.0)	140 (14.4)	
Sixties	176 (15.8)	31 (22.1)	145 (14.9)	
Seventies	145 (13.0)	20 (14.3)	125 (12.8)	
Eighties	70 (6.3)	9 (6.4)	61 (6.3)	
Sex				0.145
Male	483 (43.4)	69 (49.3)	414 (42.5)	
Female	630 (56.6)	71 (50.7)	559 (57.5)	
Pregnancy				0.787
Yes	36 (5.7)	3 (4.2)	33 (5.9)	
No	594 (94.3)	68 (95.8)	526 (94.1)	
Cigarette smoking				<0.05^a^
Never smoker	700 (62.9)	79 (56.4)	621 (63.8)	
Former smoker	325 (29.2)	53 (37.9)	272 (28.0)	
Current smoker	88 (7.9)	8 (5.7)	80 (8.2)	
Alcohol consumption				0.113
Never drinker	563 (50.6)	64 (45.7)	499 (51.3)	
Social drinker	333 (29.9)	50 (35.7)	283 (29.1)	
Consuming alcohol at least twice a week	217 (19.5)	26 (18.6)	191 (19.6)	
Underlying disease				
Hypertension				0.999
Yes	241 (21.7)	30 (21.4)	211 (21.7)	
No	872 (78.3)	110 (78.6)	762 (78.3)	
Diabetes mellitus				<0.001^a^
Yes	94 (8.4)	24 (17.1)	70 (7.2)	
No	1019 (91.6)	116 (82.9)	903 (92.8)	
Dyslipidemia				0.353
Yes	104 (9.3)	16 (11.4)	88 (9.0)	
No	1009 (90.7)	124 (88.6)	885 (91.0)	
Bronchial asthma				0.540
Yes	58 (5.2)	9 (6.4)	49 (5.0)	
No	1055 (94.8)	131 (93.6)	924 (95.0)	
Emphysema				0.999
Yes	12 (1.1)	1 (0.7)	11 (1.1)	
No	1101 (98.9)	139 (99.3)	962 (98.9)	
Myocardial infarction				0.716
Yes	18 (1.6)	1 (0.7)	17 (1.7)	
No	1095 (98.4)	139 (99.3)	956 (98.3)	
Malignancy				0.331
Yes	40 (3.6)	7 (5.0)	33 (3.4)	
No	1073 (96.4)	133 (95.0)	940 (96.6)	
Rheumatoid, collagen disease				0.716
Yes	18 (1.6)	1(0.7)	17(1.7)	0.999
No	1095 (98.4)	139(99.3)	956(98.3)	
Immunological deficiency				0.999
Yes	15 (1.3)	2 (1.4)	13 (1.3)	
No	1098 (98.7)	138 (98.6)	960 (98.7)	
Chronic kidney disease				0.999
Yes	10 (0.9)	1 (0.7)	9 (0.9)	
No	1103 (99.1)	139 (99.3)	964 (99.1)	
Neurological disease				0.706
Yes	15 (1.3)	1 (0.7)	14 (1.4)	
No	1098 (98.7)	139 (99.3)	959 (98.6)	
Vaccination prior to infection				0.543
None	214 (19.2)	87 (62.1)	127 (13.1)	
Once	18 (1.6)	6 (4.3)	12 (1.2)	
Twice	235 (21.1)	18 (12.9)	217 (22.3)	
Three times	378 (34.0)	13 (9.3)	365 (37.5)	
Four times	203 (18.2)	12 (8.6)	191 (19.6)	
Five times	65 (5.8)	4 (2.9)	61 (6.3)	
Hospitalization				<0.001^a^
Yes	204 (18.3)	97 (69.3)	107 (11.0)	
No	909 (81.7)	43 (30.7)	866 (89.0)	
Oxygen inhalation				<0.001^a^
Yes	50 (4.5)	35 (25.0)	15 (1.5)	
No	1063 (95.5)	105 (75.0)	958 (98.5)	

^a^P<0.05, indicates a statistically significant difference.

**Table II:** Symptoms during COVID-19 infection in the pre-omicron and omicron groups.

Initial symptoms during infection	Pre-omicron (%)	Omicron (%)	P-value
Fever			0.821
Yes	109 (77.9)	764 (78.5)	
No	31 (22.1)	209 (21.5)	
Cough			0.719
Yes	69 (49.3)	496 (51.0)	
No	71 (50.7)	477 (49.0)	
Fatigue			0.414
Yes	59 (42.1)	449 (46.1)	
No	81 (57.9)	524 (53.9)	
Sore throat			<0.001^a^
Yes	56 (40.0)	549 (56.4)	
No	84 (60.0)	424 (43.6)	
Dysgeusia			<0.001^a^
Yes	43 (30.7)	152 (15.6)	
No	97 (69.3)	821 (84.4)	
Headache			0.999
Yes	39 (27.9)	270 (27.7)	
No	101 (72.1)	703 (72.3)	
Dyspnea			<0.001^a^
Yes	33 (23.6)	75 (7.7)	
No	107 (76.4)	898 (92.3)	
Sputum			0.521
Yes	29 (20.7)	229 (23.5)	
No	111 (79.3)	744 (76.5)	
Joint pain			0.999
Yes	24 (17.1)	172 (17.7)	
No	116 (82.9)	801 (82.3)	
Olfactory disorder			<0.01^a^
Yes	24 (17.1)	88 (9.0)	
No	116 (82.9)	885 (91.0)	
Nasal discharge			0.102
Yes	23 (16.4)	223 (22.9)	
No	117 (83.6)	750 (77.1)	
Nasal obstruction			0.138
Yes	16 (11.4)	162 (16.6)	
No	124 (88.6)	811 (83.4)	
Sleeplessness			<0.001^a^
Yes	16 (11.4)	37 (3.8)	
No	124 (88.6)	936 (96.2)	
Myalgia			0.332
Yes	13 (9.3)	120 (12.3)	
No	127 (90.7)	853 (87.7)	
Others	12 (8.6)	62 (6.4)	
Diarrhea			0.438
Yes	10 (7.1)	54 (5.5)	
No	130 (92.9)	919 (94.5)	
Chest pain			0.114
Yes	10 (7.1)	38 (3.9)	
No	130 (92.9)	935 (96.1)	
Abdominal pain			0.361
Yes	5 (3.6)	21 (2.2)	
No	135 (96.4)	952 (97.8)	
Loss of appetite, nausea			0.052
Yes	5 (3.6)	12 (1.2)	
No	135 (96.4)	961 (98.8)	

^a^P<0.05, indicates a statistically significant difference.

**Table III:** Long COVID following COVID infection in the pre-omicron and omicron groups.

Symptom	Pre-omicron (%)	Omicron (%)	P-value
Fatigue			<0.001^a^
Yes	17 (12.1)	29 (3.0)	
No	123 (87.9)	944 (97.0)	
Alopecia			<0.001^a^
Yes	11 (7.9)	10 (1.0)	
No	129 (92.1)	963 (99.0)	
Loss of concentration			<0.01^a^
Yes	10 (7.1)	23 (2.4)	
No	130 (92.9)	950 (97.6)	
Dyspnea			<0.01^a^
Yes	10 (7.1)	20 (2.1)	
No	130 (92.9)	953 (97.9)	
Olfactory disorder			<0.001^a^
Yes	10 (7.1)	3 (0.3)	
No	130 (92.9)	970 (99.7)	
Dysgeusia			<0.001^a^
Yes	9 (6.4)	9 (0.9)	
No	131 (93.6)	964 (99.1)	
Muscle weakness			<0.05^a^
Yes	8 (5.7)	19 (2.0)	
No	132 (94.3)	954 (98.0)	
Memory disorder			<0.05^a^
Yes	6 (4.3)	14 (1.4)	
No	134 (95.7)	959 (98.6)	
Headache			<0.05^a^
Yes	6 (4.3)	11 (1.1)	
No	134 (95.7)	962 (98.9)	
Cough			0.116
Yes	6 (4.3)	19 (2.0)	
No	134 (95.7)	954 (98.0)	
Sputum			0.095
Yes	5 (3.6)	15 (1.5)	
No	135 (96.4)	958 (98.5)	
Sleeplessness			0.079
Yes	5 (3.6)	14 (1.4)	
No	135 (96.4)	959 (98.6)	
Chest pain			<0.05^a^
Yes	4 (2.9)	4 (0.4)	
No	136 (97.1)	969 (99.6)	
Palpitations			0.691
Yes	4 (2.9)	9 (0.9)	
No	136 (97.1)	964 (99.1)	
Depressed mood			0.093
Yes	3 (2.1)	6 (0.6)	
No	137 (97.9)	967 (99.4)	
Muscle pain			0.364
Yes	2 (1.4)	8 (0.8)	
No	138 (98.6)	965 (99.2)	
Joint pain			0.999
Yes	1 (0.7)	8 (0.8)	
No	139 (99.3)	965 (99.2)	
Diarrhea			0.999
Yes	0 (0.0)	4 (0.4)	
No	140 (100.0)	969 (99.6)	
Abdominal pain			0.999
Yes	0 (0.0)	2 (0.2)	
No	140 (100.0)	971 (99.8)	

^a^P<0.05, indicates a statistically significant difference.

**Table IV:** Factors related to long COVID following infection with COVID-19.

Factor	No LC, n=949 (%)	LC, n=164 (%)	P-value
Age group			<0.001^a^
Teens	101 (10.6)	3 (1.8)	
Twenties	124 (13.1)	13 (7.9)	
Thirties	121 (12.8)	17 (10.4)	
Forties	150 (15.8)	25 (15.2)	
Fifties	128 (13.5)	40 (24.4)	
Sixties	147 (15.5)	29 (17.7)	
Seventies	119 (12.5)	26 (15.9)	
Eighties	59 (6.2)	11 (6.7)	
Sex			0.932
Male	411 (43.3)	72 (43.9)	
Female	538 (56.7)	92 (56.1)	
Pregnancy			0.339
Non-pregnancy	505 (53.2)	89 (54.3)	
Pregnancy	33 (3.5)	3 (1.8)	
Smoking status			0.106
Never smoker	606 (63.9)	94 (57.3)	
Former smoker	271 (28.6)	54 (32.9)	
Current smoker	72 (7.6)	16 (9.8)	
Alcohol consumption			0.743
Never drinker	482 (50.8)	81 (49.4)	
Social drinker	277 (29.2)	56 (34.1)	
Consuming alcohol at least twice a week	190 (20.0)	2 7 (16.5)	
Vaccination status			<0.001^a^
0-1 times	171 (18.0)	61 (37.2)	
2-6 times	778 (82.0)	103 (62.8)	
Underlying disease			<0.001^a^
None	536 (56.5)	68 (41.5)	
Any	413 (43.5)	96 (58.5)	
Antiviral drug use			0.131
Yes	95 (10.0)	23 (14.0)	
No	854 (90.0)	141 (86.0)	
Hospital admission			<0.001^a^
Admission	138 (14.5)	66 (40.2)	
No admission	811 (85.5)	98 (59.8)	
Oxygen inhalation			<0.001^a^
Oxygen required	28 (3.0)	22 (13.4)	
No oxygen demands	921 (97.0)	142 (86.6)	
Omicron variant			<0.001^a^
Non-omicron	88 (9.3)	52 (31.7)	
Omicron	861 (90.7)	112 (68.3)	

^a^P<0.05, indicates a statistically significant difference. LC, long COVID.

**Table V:** Factors related to long COVID following infection with COVID-19 in the multivariate logistic regression analysis.

		CI		
Factor	OR	2.5%	97.5%	P-value
Admission	1.950	1.220	3.120	<0.01^a^
Diabetes mellitus	1.110	0.626	1.950	0.729
Oxygen inhalation	1.290	0.626	2.580	0.470
Age	1.160	1.050	1.280	<0.01^a^
Vaccination	0.831	0.729	0.948	<0.01^a^
Omicron	0.506	0.301	0.851	<0.01^a^

^a^P<0.05, indicates a statistically significant difference. OR, odds ratio; CI, confidence interval.

**Table VI:** Factors related to long COVID following infection with COVID-19 in the omicron group.

Factor	No LC, n=861 (%)	LC, n=112 (%)	P-value
Sex			0.839
Male	365 (42.4)	49 (43.8)	
Female	496 (57.6)	63 (56.3)	
Pregnancy			0.567
Non-pregnancy	465 (54.0)	61 (54.5)	
Pregnancy	31 (3.6)	2 (1.8)	
Smoking status			0.669
Never smoker	554 (64.3)	67 (59.8)	
Former smoker	240 (27.9)	32 (28.6)	
Current smoker	67 (7.8)	13 (11.6)	
Alcohol consumption			0.513
Never drinker	440 (51.1)	59 (52.7)	
Social drinker	245 (28.5)	38 (33.9)	
Consuming alcohol at least twice a week	176 (20.4)	15 (13.4)	
Vaccination status			0.999
0-1 times	123 (14.3)	16 (14.3)	
2-6 times	738 (85.7)	96 (85.7)	
Underlying disease			<0.001^b^
None	496 (57.6)	42 (37.5)	
Any	365 (42.4)	70 (62.5)	
Hospital admission			<0.01^a^
Admission	84 (9.8)	23 (20.5)	
No admission	777 (90.2)	89 (79.5)	
Oxygen inhalation			0.0833
Oxygen required	11 (1.3)	4(3.6)	
No oxygen demands	850 (98.7)	108 (96.4)	

^a^P<0.05 and

^b^P<0.01, indicates a statistically significant and highly statistically significant difference, respectively. LC, long COVID.

**Table VII:** Associations between underlying diseases and long COVID following infection with COVID-19 in the omicron group in the multivariate logistic regression analysis.

Underlying disease	OR	2.5% CI	97.5% CI	P-value
Emphysema	6.66	2.088	7.999	<0.001^a^
Bronchial asthma	5.13	2.386	5.465	<0.001^a^
Rheumatoid, collagen disease	3.31	1.232	5.606	<0.05^a^
Chronic kidney disease	2.22	0.566	6.695	0.312
Hypertension	1.77	1.135	2.375	<0.01^a^
Malignancy	1.75	0.765	3.399	0.222
Dyslipidemia	1.67	0.945	2.558	0.088
Myocardial infarction	1.67	0.546	4.388	0.424
Diabetes mellitus	1.66	0.896	2.676	0.125
Immunological deficiency	1.40	0.371	4.863	0.660
Neurological disease	0.59	0.093	4.113	0.606

^a^P<0.05, indicates a statistically significant difference. OR, odds ratio; CI, confidence interval.

## Data Availability

The data generated in the present study may be requested from the corresponding author. The data are not publicly available due to privacy or ethical policy.
